# Comparing the Anthropometrics, Body Composition, and Strength Performance of Male and Female Italian Breaking Athletes: A Pilot Study

**DOI:** 10.3390/sports12070197

**Published:** 2024-07-22

**Authors:** Bruno Ruscello, Gabriele Morganti, Antonio De Fano, Flavio Mancina, Laura Lunetta, Giuseppe Di Mauro, Claudio Cogoni, Edilio Pagano, Nicolò Marco Brigati, Andrea Di Castro, Antonio Gianfelici, Raffaella Spada, Elvira Padua, Chiara Ragona

**Affiliations:** 1 Department of Human Sciences and Promotion of the Quality of Life, San Raffaele Roma Open University, 00166 Rome, Italy; gabriele.morganti@uniroma5.it (G.M.); elvira.padua@uniroma5.it (E.P.); 2 Italian DanceSport and Musical Sports Federation (FIDESM), 00135 Rome, Italy; antonio.defano@unich.it (A.D.F.); flavio.mancina@gmail.com (F.M.); presidente@fids.it (L.L.); kacyodeklan@gmail.com (G.D.M.); ormusforce@libero.it (C.C.); pagano@fids.it (E.P.); 3 School of Sport and Exercise Sciences, Faculty of Medicine and Surgery, Tor Vergata University, 00133 Rome, Italy; 4 ASD Luiss SportLab, 00197 Rome, Italy; 5 Behavioural Imaging and Neural Dynamics (BIND) Center, Department of Medicine and Aging Sciences, University “G. D’Annunzio” of Chieti-Pescara, 66100 Chieti, Italy; 6 Sport Medicine and Science Institute “A. Venerando”, Italian Olympic National Committee (CONI), 00197 Rome, Italy; ext_nicolo.brigati@coni.it (N.M.B.); ext_andrea.dicastro@coni.it (A.D.C.); ext_antonio.gianfelici@coni.it (A.G.); ext_raffaella.spada@coni.it (R.S.); ext_chiara.ragona@coni.it (C.R.)

**Keywords:** breaking, anthropometric measures, body composition, somatotype, strength performance, male sport, female sport, Olympic sport, urban sport

## Abstract

Breaking is a performative art that has recently undergone a process of sportification, developing into an aesthetic sport included in the 2024 Paris Olympic Games. Despite its growing worldwide popularity, there is a lack of research on Breaking. Accordingly, this pilot study’s aim was twofold: (a) to provide an initial understanding of the anthropometric measures, body composition data, somatotype profiles, and strength performance of male (B-boys) and female (B-girls) Italian Breakers divided into elite (international) and sub-elite (national) levels and (b) to guide further research on the area, providing the methodological approach for future investigations. A total of 24 B-boys (elite *n* = 5; sub-elite *n* = 19) and 9 B-girls (elite *n* = 3; sub-elite *n* = 6) were included in this study. Descriptive analyses revealed that B-boys and B-girls displayed low height and weight (1.70 m (63.8 kg) and 1.58 m (54.2 kg), respectively), low levels of body fat percentages (10.3% and 17.6%, respectively), and a balanced mesomorph somatotype (2.28–4.64–2.69 and 2.34–5.16–2.38, respectively), revealing a marked development of muscular mass. Due to the small sample size, Welch’s test and correlation analyses did not report any elite vs. sub-elite difference. It was hypothesized that Breakers’ morphological profiles result from the selection procedures and training regimens related to Breaking aesthetic, athletic, and physiological demands.

## 1. Introduction

Breaking is a performative art form that originated from the African American and Caribbean communities of New York City (USA) in the late 1960s [1]. It underwent a process of sportification, which started in the 1980s when it gained followers all over the world as proper sports competitions with dedicated judging panels and cash prizes awarded to victors began to be organized regularly and continued through the 1990s when, following its rise in popularity, it became part of the International Sports Dance Federation (WSDF). In Italy, it is currently a recognized and regulated DanceSport affiliated with the Italian DanceSport and Musical Sports Federation (FIDESM) [2]. Following its debut at the Youth Olympic Games in Buenos Aires in 2018, which received great acceptance, in 2019, the International Olympic Committee (IOC) announced that Breaking would be the first DanceSport to participate as an Olympic sport, being added to the Olympic program for the 2024 Summer Games in Paris [3,4].

Breaking’s transition from performing arts to Olympic sports introduced new considerations for designing effective training programs [5] and talent identification and development systems [6,7]. Indeed, nations compete indirectly for collective national success, generally measured by the medal tally at Olympic Games [8]. As such, a vast body of knowledge has been produced on finding the right strategic approach to ensure constant and sustainable high-performance outcomes, such as recruitment, selection, and development of talented youth athletes to support them throughout the youth-to-senior transition [9]. There is indeed an increasing need to provide sporting practitioners with reliable scientific information, to assist and inform their training proposals and selection procedures, and to help them compete at the highest levels. However, Breaking is still underexplored scientifically, and data on Breakers are limited. There are few published studies about their morphological characteristics [10,11,12], physiological traits, biomechanical profiles, and strength outputs [11] and no studies that explored their somatotypes.

Past research has investigated physical attributes that may positively interact with expertise development in sporting contexts to guide practitioners’ and selectors’ decision-making [13,14]. In line with this, several studies have reported different anthropometric values, somatotype, and body composition data between successful and unsuccessful athletes [15,16,17]. Moreover, it has also been acknowledged that there are both intra- and inter-sports variations in and preferences for ideal body proportions and favored body composition data [12,18,19,20]. Indeed, the standard of beauty may influence parents’ enrolment decisions on their children’s sports participation, training practices (i.e., how a sport is perceived and taught), ways of being and doing in the sport in question (i.e., style of play in team sport and choreography selection in DanceSport), and coaches’ selection procedures (i.e., talent pathway) [21]. In the case of aesthetic sports, such as Breaking, physical attributes and body shape may be even more important, given their influence on judges’ evaluation [22], as well as on the biomechanics of technical movements [23,24], thus constraining choreography selection and indirectly influencing competition results and performance outcomes [25].

In dance, some research has investigated dancers’ height and weight characteristics [25], as well as body composition [10,26] and somatotype [27,28]. In a comparison between Ballet, Contemporary Dance, and DanceSport (i.e., unspecified couple dance), Liiv and colleagues [28] showed that DanceSport dancers are taller and heavier, less muscular, and with greater adiposity compared to Ballet dancers. Moreover, another study conducted by Liiv et al. [25] revealed that (a) according to dance style (Standard, Latin American, and Ten Dance), dancers have different anthropometric profiles and somatotypes, and (b) DanceSports require a different somatotype compared to other aesthetic sports, as the authors reported that DanceSport dancers were found to be less mesomorphic (i.e., relative musculoskeletal robustness) and more ectomorphic (i.e., relative linearity) than gymnasts, Ballet dancers, and figure skaters.

In the context of Breaking, a few studies revealed that Breaker-boys (B-boys) and Breaker-girls (B-girls) are on average shorter than other DanceSport, Contemporary, and Latin American dancers [10,12] and show similar body fat percentages to Ballet dancers [9]. Moreover, Arundale et al. [12] reported that Breakers display lower-limb asymmetries and proposed that this is due to the specific training regimens they are generally submitted to (i.e., absence of generic strength training vs. prevalence of specific Breaking training). The authors also recorded high levels of hip strength variability and presented that this is due to personal preferences and characteristics (Breakers who favor power moves and the ones who instead prefer top rock and/or footwork steps [12]). These suggest that training practices, athletic demands, and selection procedures favoring specific body proportions and beauty standards cause differences in athletes’ anthropometric measures, body composition data, biomechanics, and physiological characteristics [28,29,30]. However, considering the limited data found in the literature for Breaking, no studies have specifically investigated B-boys’ and B-girls’ attributes (i.e., body shapes, compositions, and biomechanical, physiological, and strength profiles) to explore eventual differences and similarities with other DanceSports or aesthetic sports and whether these may be linked to higher achievements in the sport in question, thus indicating the need for targeted exploration to be conducted.

Accordingly, this cross-sectional and retrospective observational pilot study has a twofold aim: first, to provide an initial exploration and understanding of the anthropometric measures, body composition data, somatotype profiles, and strength performance of male and female Italian Breakers competing at the elite (international) and sub-elite (national) levels and, second, to guide further research in this area, providing the methodological approach for future investigations.

## 2. Materials and Method

### 2.1. Study Design

This pilot study employed a cross-sectional and retrospective observational research design, analyzing a sample of Italian Breakers (both male and female) competing at the national and international levels.

This pilot study investigated the following dependent variables: anthropometric measures, including body height and weight, body mass index (BMI), eight skinfold measures (subscapular, triceps, midaxillary, chest, suprailiac, abdominal, thigh, and calf), circumferences of the upper and lower limbs (biceps, thigh middle, and calf), and bone breadths (humerus and femur), and body composition values, including calculations of percentages of body fat (%), body fat mass (kg), body fat-free mass (kg), and somatotype indices. This pilot study also considered strength performance outcomes obtained from physical fitness tests: jump testing to explore vertical jump height and lower-limb power output and the Power Push-Ups (PPUs) test to determine the upper-body power output.

The independent variables considered in this pilot study were gender and levels of qualification (i.e., elite and sub-elite).

### 2.2. Subjects

Thirty-three Italian Breaking athletes volunteered to participate in this pilot study. The sample included 24 B-boys [age 24.2 (4.8) years; weight 63.8 (6.6) kg; height 1.71 (5.7) m; BMI 21.9 (2.2) kg·m^−2^] and 9 B-girls [age 21.9 (5.5) years; weight 54.2 (6.3) kg; height 1.58 (7.7) m; BMI 21.5 (1.7) kg·m^−2^]. The pilot study population is the Italian Breaking athlete community, which currently consists of about 800 members, so the study sample represents about 4.1% of the population. To be admitted into this pilot study, reference was made to the national and international qualification rankings, compiled by the FIDS and the World DanceSport Federation (WDSF), respectively. The final selection was carried out by the technical staff of the Italian Breaking National Team. The selected sample was then further subdivided into elite and sub-elite groups, both male [elite *n* = 5; sub-elite *n* = 19] and female [elite *n* = 3; sub-elite *n* = 6]. This division was carried out by the coaching panel based on their participation in or absence from international competitions as representatives of the Italian national team. In Table 1, the relative frequency data of distribution in elite and sub-elite groups, categorized by gender, are presented.

The Breakers had a minimum of 3–8 years of experience at competitive levels (sub-elite at the national level and elite at the international level) and engaged in a minimum of 4 up to 10 training sessions per week for the development of specific fitness. Power, agility, and repeated explosive activities (REAs) have been consistently incorporated into their training regimen, particularly during the competitive season, which is the period under investigation in this paper.

Participation in this pilot study was restricted to subjects who met the following inclusion criteria: (1) regular training and competitive participation during the competitive season at both national and international levels, (2) best ranking in the national and international rankings, and (3) possession of a valid medical certificate. All subjects were deemed healthy and free of any drug consumption. This retrospective study was approved by the Institutional Review Board (IRB) of the University of Rome “San Raffaele” through the appropriate Ethical Committee (Comitato Etico Territoriale Lazio Area 5), with the protocol number ifo_058.IFO_AOO.REGISTRO UFFICIALE.0002848.26-02-2024—Parere Studio IB (Italian Breaking)—Registro Sperimentazioni N. 95/IRE/24.

All participants provided written informed consent after being comprehensively informed about the benefits and potential risks associated with the study procedures. They were explicitly made aware of their right to withdraw from this study at any time without penalty. All research procedures adhered strictly to the Declaration of Helsinki of the World Medical Association for the conduct of clinical research.

### 2.3. Anthropometric Measures

Body height (m) and body mass (kg) were measured to the nearest 0.1 cm (reported in m) and 0.1 kg, respectively. Both height and weight measurements were conducted in the morning at approximately 30 min post-awakening, fasting before breakfast. The body mass index (BMI) was calculated from the body height and weight as follows: weight/height^2^. Skinfold measurements were taken from eight sites (subscapular, triceps, midaxillary, chest, iliac crest, abdominal, thigh, and calf skinfolds) to the nearest 0.1 mm [20]. Bicep girth (cm) was measured with the arm in a tensed position; thigh middle girth (cm) was recorded with athletes standing erect with their weight evenly distributed on both feet and legs slightly parted, while calf girth (cm) was measured with athletes sitting at the end of a table, having their legs hanging. Humerus and femur breadths were measured to the nearest 0.1 mm (reported in cm). All the measurements were conducted on the right side of the body, according to standardized procedures [31].

Three series of anthropometric measurements were taken for each presented site, and the mean was recorded. All skinfold measurements were obtained indoors at approximately the same time of the day. Skinfold thicknesses were measured using a Holtain (Crymmich, UK) skinfold caliper. Girths were calculated using a common tape measure.

### 2.4. Body Composition

The sum of seven skinfolds (7SKFS) (subscapular, triceps, midaxillary, chest, iliac crest, abdominal, and thigh) was used for the determination of body density, using the Jackson and Pollock equation [32]. Body fat percentage (%) was then calculated with the equation of Siri [33]. Body fat mass (kg) was derived accordingly, while body fat-free mass (kg) was calculated from the body mass and body fat mass.

Somatotype components (endomorphy–mesomorphy–ectomorphy) were calculated according to the Carter and Heath [34] anthropometric somatotyping method.

### 2.5. Strength Performance

To determine the lower-limb muscle power output, a repetitive countermovement jump (CMJ5) test was used. The CMJ5 test explains the explosive elastic force which is defined as the ability to achieve a considerable expenditure of strength during a muscle stretching/shortening cycle, that is, during an eccentric contraction immediately followed by a concentric contraction [35,36,37].

According to the instructions of the CMJ5 test protocol, participants were required to maintain an upright position, rapidly flexing the hips, knees, and ankles before jumping. Countermovement depth was not controlled. The participants completed 2 sets of multiple CMJs with arm swings, aiming to jump as high as possible for 5 consecutive efforts without a pause between jumps [38]. To mitigate the negative effects of fatigue, a pause of approximately 3 min was implemented between sets [39].

The CMJ5 test was conducted using the Optojump photocell system (Microgate, Bolzano, Italy) [40]. Data recorded (jump flight time, jump contact time, jump height, and power output) were obtained by the best average score over the three central CMJs between the two sets performed. Additionally, the best values for each variable of study were also recorded and compared to the mean values to obtain the coefficient of variation (%).

To determine the upper-body muscle power, the Power Push-Ups (PPUs) test was adopted [41]. Participants began the push-ups in the prone-up position with their arms extended, forearms and wrists in pronation, and feet at a biacromial width (shoulders). Their hands were placed approximately shoulder-width apart at a self-selected, comfortable distance. In the standard position, the toes served as the pivot point, with knees straight and feet together. The exercise was performed on a tartan surface without the use of a mat.

To ensure the accurate execution and control of hand positions, a bench step of 15 cm was used as a support column for both hands. This setup was implemented to maximize the precision of measurements with the encoder, minimizing interference during the eccentric phase. It ensured that the encoder followed the correct path during the execution process [37].

In the prone-up position, the subject executed a rapid eccentric countermovement until their chest was positioned just above the bench step. This was followed by an explosive concentric push-up. The sequence was repeated for five trials at the maximum possible speed, ensuring consistent hand placement to maintain the same movement amplitude.

Participants were instructed to maintain a tight torso, ensuring that the shoulders, hips, knees, and ankles formed a straight line throughout the push-up. They were advised not to pause at the top, keep their elbows flexed at approximately 90°, and lower the chest nearly to the height of the bench floor [41,42].

Power outputs were collected using a linear encoder [43,44,45,46]. The encoder (ET-Enc-02, Ergotest Technology AS, Porsgrunn, Norway) was attached at the sternocostal notch via a collar when performing a push-up, directly vertical to the ground, without disturbing the push-up depth. The linear encoder measured with a resolution of 0.019 mm and counted the pulses with a 5 ms interval vertical displacement in relation to the lowest point of the barbell or person (zero distance).

The testing procedures of strength performance (both lower and upper body) were preceded by a 15 min warm-up that included running indoors for 5 min at the pace chosen by the subjects, followed by plyometric drills for the lower and upper body and refamiliarization with the test exercises, with an explanation and demonstration of the proper technique to perform the movement [46]. Participants were already familiar with the testing protocols and, before recording, were instructed to perform as many trials as necessary, until they were comfortable with the movements [12].

### 2.6. Data Analysis

The data are presented as mean and standard deviation [M(SD)] along with 95% confidence intervals (95% CIs). The normality assumption was assessed using the Shapiro–Wilk test. Intraclass correlation coefficients (ICCs) for average measures were used as indices of relative reliability. An intraclass correlation coefficient (ICC) exceeding 0.70 is commonly deemed acceptable when utilizing a measurement instrument with groups of participants [47]. To investigate potential differences between genders and among elite and sub-elite athletes, non-parametric independent *t*-tests were utilized (Welch’s t).

Welch’s *t*-test is useful for comparing the means from small, unequal-sized samples without assuming equal variances, as in our pilot study. It handles the issue of unequal variances well, often providing robust results [48].

**Figure 1:**
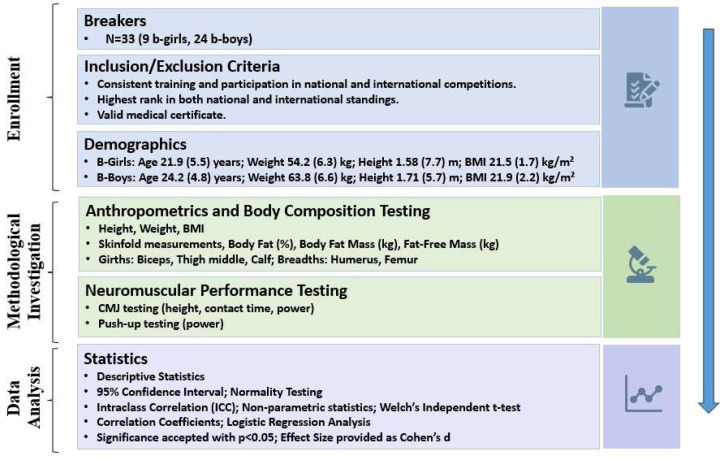
Flow diagram illustrating the enrollment, methodological investigation, and analyses of the Breakers involved in this research project.

In addition to null hypothesis testing, the effect size (ES) as Cohen’s d was reported. Absolute ES values of 0.20, 0.50, 0.80, and >1 represented small, medium, large, and very large effects, respectively. To calculate possible associations between variables, the Bravais–Pearson correlation coefficient (r) was computed. To uncover potential factors predicting the attained qualification level (elite vs. sub-elite), where the qualification level is the dependent variable, the measurement variables were regarded as independent and incorporated into a logistic regression analysis. Corresponding *p*-values are provided for each analysis, with statistical significance set at *p* < 0.05. IBM SPSS 27 for Windows (SPSS Inc., Chicago, IL, USA) and Jamovi (https://www.jamovi.org/, accessed on 10 January 2024) were utilized to analyze and process the collected data. A flow-chart diagram summarizing the study protocol is provided in Figure 1.

## 3. Results

### 3.1. Anthropometric Measures

Table 2 separately displays male and female Italian Breakers’ bodily dimensions of weight and height. As one might hypothesize, they significantly differed between genders. Nevertheless, these variations result in practically identical body mass index (BMI) values, even when considering the 95% confidence interval of the mean, confirming nearly equal ratios between weight and the square of height in both genders.

To examine potential differences in measurements between elite and sub-elite groups, independent sample Welch’s *t*-tests were applied separately for males and females. In the male group, no significant differences were observed in the measured variables (*p* > 0.05), with Cohen’s d values (height, weight, and BMI) of −0.316, 0.408, and 0.687, respectively. However, a significant difference was found in body weight among females (*p* = 0.026; Cohen’s d = 1.816). Nevertheless, given the small sample size, this finding should be interpreted with caution.

Table 3 presents descriptive statistics and the results of Welch’s *t*-tests conducted to compare skinfold measurements, body girths, and breadths between genders. To ensure the reliability of the skinfold measurements, an intraclass correlation coefficient (ICC) was calculated, yielding a high reliability (ICC = 0.992; 95% CI = 0.872–0.957; *p* < 0.001). Statistically significant gender differences were observed for almost all skinfold measurements, bicep girths, and body breadths. However, no differences were noted in the subscapular, thigh, and calf girths.

To investigate the differences in measurements between elite and sub-elite athletes, Welch’s *t*-tests were conducted. No statistically significant differences were found within the male athletes’ group (*p* > 0.05; Cohen’s d values ranging from −0.763 for the subscapular to 0.021 for the midaxillary regions). In the female group, statistically significant differences were observed for thigh girths (*p* < 0.001, d = 4.227) and calf girths (*p* = 0.050, d = 1.785). However, despite their significance, these findings should be interpreted with caution due to the small sample size.

### 3.2. Body Composition

Table 4 provides body composition values along with relevant descriptive statistics and Welch’s *t*-test results between genders.

To identify potential differences in these measurements between elite and sub-elite athletes, an independent sample Welch’s *t*-test was conducted. No statistically significant differences were found between elite and sub-elite male athletes (*p* > 0.05). Effect sizes, calculated as Cohen’s d, varied across measurements: −0.05 for body mass percentage, 0.10 for body fat mass, and 0.49 for fat-free body mass. In the female group, significant differences were observed in relation to body fat-free mass (kg) measurements (*p* = 0.02; d = 2.24). However, given the small sample size (*n* = 9), these findings should be interpreted with caution.

Table 5 separately displays male and female Italian Breakers’ somatotype profiles, along with Welch’s *t*-test results between genders. Indeed, both sexes were composed of a balanced mesomorph somatotype.

To identify potential differences in these measurements between elite and sub-elite athletes, Welch’s *t*-tests were conducted.

No statistically significant differences were found within the male group. However, effect sizes, calculated as Cohen’s d, indicated notable variations across body types: 1.05 for endomorphic, 0.40 for mesomorphic, and −0.74 for ectomorphic, suggesting differential impacts on these traits.

Significant differences were observed in the female group regarding ectomorphic body types (*p* = 0.05; d = −1.45). However, given the small sample size (*n* = 9), these findings should be interpreted with caution.

### 3.3. Strength Performance

In Table 6, descriptive statistics for vertical force assessments of the lower and upper limbs, conducted through CMJ (countermovement jump) and push-up tests, are presented. To identify potential differences in performance metrics between elite and sub-elite athletes, independent sample Welch’s *t*-tests were conducted. No statistically significant differences emerged within the group of male athletes. However, the effect sizes, calculated as Cohen’s d, varied across measurements. For example, for the CMJ5 test, the peak height showed an effect size of 0.40, the mean height was 0.28, the best contact time was 0.34, the mean contact time was 0.33, the peak power was 0.17, and the mean power was −0.66; the push-up power recorded an effect size of 0.17. Similarly, in the female group, no significant differences were observed. Nevertheless, the effect sizes varied: the CMJ5 peak height was 0.72, mean height was 0.61, best contact time was 0.68, mean contact time was −0.04, peak power was 0.44, mean power was 0.47, and push-up power was 0.96. These effect sizes, particularly the moderate and large ones, suggest that there may be specific physiological or biomechanical traits that differentiate higher-performing athletes from their less elite counterparts. These traits can be targeted in training programs to enhance performance.

### 3.4. Associations among the Variables under Consideration: Correlations and Logistic Regression Analysis

To calculate the possible associations between variables, both the Bravais–Pearson correlation coefficients (r) and Spearman’s rho were computed. The relevant results are provided in the Supplementary Materials: Table S1 for males and Table S2 for females. Nevertheless, given the small sample size, these analyses must be interpreted with caution.

To identify potential predictors of the achieved qualification level, which was set as the dichotomous dependent variable (elite vs. sub-elite), measurement variables were treated as independent and included in a logistic regression analysis. However, no variable or combination of variables provided odds ratios significant enough to identify valid predictors; the Wald tests did not reach statistical significance (*p* > 0.05), and the 95% confidence intervals for the calculated Exp(B) all included the value of 1.

## 4. Discussion

To our knowledge, this study was the first that aimed to assist professionals in the field of Breaking, whose significance on a global scale is evidenced by the inclusion of this dance discipline in the upcoming 2024 Olympic Games in Paris. In more detail, this pilot study provided the first exploration into the anthropometric measures, body composition data, and strength performance of male and female Italian elite (international) and sub-elite (national) Breakers. Overall, the findings revealed that B-boys and B-girls display low height and weight, low levels of body fat percentages, and a balanced mesomorph somatotype, indicating a marked development of muscular mass.

The BMIs of male and female international and national Italian Breakers are within the normal range when compared to the general population with normal weight (BMI = 18.5–25 kg·m^−2^) and similar to those reported by Montalbán-Méndez and colleagues [10], who recorded the BMI values of Spanish international Breakers. Moreover, both male and female Italian Breakers were found to be shorter in comparison to other dancers specialized in other DanceSport, Ballet, and Latin American Dance activities [25,28] but higher when compared to Italian senior gymnasts [49]. The lower height recorded by Italian Breakers may be attributed to the fact that this type of dance does not require participants to perform choreography that includes lifts, which require the female dancer to be lifted above the partner’s head, such as the case for Ballet and DanceSport athletes [28], and which therefore require dancers to have greater limb length, as well as higher trunk height values [25], eventually linked to ideals of effortless grace [50]. In contrast, Breaking movements are characterized by *transitions*, also referred to as *links*, which support B-boys and B-girls in changing from one position to another. The most basic *link* happens when the Breaker transitions from a standing position (*top rock*) to a ground position (*down rock*), and it is generally referred to as *get down* [51]. For example, the *drop* is a short transition used to reach the floor creatively, following the rhythm of the music [52], and requires body control and balancing skills. A recent biomechanical simulation conducted on gymnastics suggested how increasing body height may make balancing more difficult and indicated how males with short/normal bodies and females with short bodies may have an innate ability to perform the *planche* (an iconic gymnastics movement considered one of the most required skills in this discipline) [53]. Moreover, research has shown that smaller gymnasts are also favored when performing movements that include whole-body rotations [23,53], and indeed, Claessens et al. [22] revealed how in world-class gymnasts, lower-ranking female athletes were on average 6.5 cm taller in comparison to their higher-ranking counterparts, thus indicating how having ideal body proportions for a given sport plays an important role in defining talent developmental opportunities, ultimately shaping performance outcomes. In line with this, Breakers’ lower height may favor their *top rock-to-down rock* transition, and consequently, the anthropometric measures recorded in our study may be due to selection biases that favor shorter individuals. Shorter Breakers may also be favored when performing a *flare*, which is a skill that involves body rotation and dynamic balance, requiring having hands on the floor while the legs are moving (i.e., similar to the pommel horse performed by gymnasts).

The results from the body composition analysis revealed that Italian B-girls recorded lower percentages of body fat compared to other DanceSport and Contemporary dancers [28]. Indeed, Italian B-girls recorded similar percentages of body fat to female Ballet dancers (17.54% vs. 17.52%) [28]. In line with this, Italian B-boys showed lower values of body fat compared to their counterparts involved in DanceSport, Contemporary, and Ballet activities [28]. Similarly, the somatotype analysis revealed how both Italian B-boys and B-girls have the balanced mesomorph somatotype, showing lower ectomorph and endomorph values, thus indicating their marked development of muscular mass. This somatotype profile has been already associated with high-level world-class female gymnasts [22] and Latin American dancers [25]. In gymnastics events, athletes perform many dynamic and static movements in combination and are also required to incorporate aerial phases [54,55], while Latin American dancers are required to exhibit fast leg movements and systematic turns during their choreography [25]. Both activities can therefore be facilitated when participants display lower levels of body fat and higher mesomorphic values, both associated with a greater level of muscularity. In Breaking, once Breakers are in *down rock*, they generally perform *footwork steps*, which are movements performed on the floor with different body supports (i.e., hands, feet, knees) [52,56]. Moreover, Breakers perform *acrobatic movements*, which involve no contact with the ground having a defined aerial phase, and *power moves*, which are defined as explosive movements conducted in dynamic equilibrium that include rotation on more than one axis (i.e., sagittal, longitudinal, and transverse), performed on four, three, two, or just one support [56]. Research conducted on time–motion analysis in Breaking revealed how Battles are short, lasting on average 182 s, thus concentrating a high level of effort in a short period of time, resonating with the rate of exertion generally seen in climbing, gymnastics, or martial arts activities [52]. In their study, Gutiérrez-Santiago and colleagues [55] highlighted that Breakers spend a high amount of time performing *power moves* and *acrobatics*, which are actions that require explosiveness, dynamic strength, and muscular power and which may also include high-intensity isometric holds, which can be demanding for the cardiovascular system [57]. This tendency towards the utilization of highly physically demanding movements in Breaking Battles has been recently confirmed by a more recent and in-depth time–motion analysis that investigated a total of 142 international Battles [52]. As such, the greater levels of muscularity, associated with lower levels of body fat percentages and the balanced mesomorph somatotype, recorded in our study which characterizes the Italian B-boys and B-girls population may be due to Battles’ physical and muscular demands and therefore resultant of selection procedures, training regimens, and regular ways of doing things (i.e., Breaking practices).

Pérez-Portela et al. [52] suggested how Breaking is evolving into a more and more aesthetic sport. Breakers are willing to add further content and movements to their rounds to positively impress the judges. In line with this, Battles are increasing in duration and developing into even more demanding contests in terms of both athletic needs and physical efforts. Consequently, to competitively conform to the Battles’ performance model and their increasing aesthetic needs and duration, Breakers are required to possess high levels of muscular strength to perform explosive movements aimed at impressing judges and to delay the arrival of fatigue and its detrimental effects on muscular strength. As such, investigating Breakers’ neuromuscular performance may be the required next step to better define and guide training procedures. Indeed, Claudino et al. [58], in their meta-analysis conducted on the efficacy of CMJ to monitor neuromuscular performance, concluded by indicating the importance of considering average values when reporting CMJ test results. The authors proposed how this would guarantee more sensitive monitoring of the effects of fatigue and training super-compensation on performance. Similarly, considering that Breakers’ movements are also characterized by their ability to maintain position supporting the weight of the body with their upper limbs (i.e., when they perform *power moves* and/or are in *down rock* position performing *footwork steps*), investigating, monitoring, and training their trunk and upper-limb strength outputs may be essential. Importantly, the strength performance investigations conducted in this study did not reveal any differences between sub-elite- and elite-level Italian Breakers. It is hypothesized that this was due to the small sample size. However, it is worth considering here that Arundale and colleagues [12] proposed that the investigation of Breakers’ vertical force production, a different jump test other than the CMJ, may be more appropriate to assess their sport-specific demand. Nevertheless, the results obtained in this study may act as a blueprint and reference values for future investigations on this research area.

Breaking promotes peer-to-peer interactions and teaching and the self-exploration of new movements and techniques [59]. This may help create collective ideals related to successful aesthetics and beauty standards [21], linked to ideals of body proportions and shapes, as well as to a defined fashion style. Indeed, being part of a specific social group, such as in the case of Breaking, means positively corresponding to customs and behaviors to fit in with the requirements of the given organizational structure and conforming to standards which may result in functionally satisfying Breaking athletic, physical, and aesthetical needs (i.e., short body height and balanced mesomorph, e.g., [59]). Therefore, the anthropometric measures and body composition data in the male and female Italian Breakers population, revealed in our pilot study, may be due to the socio-cultural and historical roots of Breaking and how they influenced its developmental trajectory and competition format, having ultimately shaped its specific physiological, athletic, and physical demands.

### 4.1. Practical Implications and Future Directions

This study offers useful insights that can have direct applications in the development and training of B-boys and B-girls. The identification of common physical characteristics among top performers can guide trainers and coaches in designing specialized and individualized training regimens that enhance these traits. Indeed, findings obtained from this study can contribute to the optimization of training strategies, ensuring they are tailored to exploiting the inherent strengths and improving the potential weaknesses identified in Breakers. This could eventually lead to improved performance in competitions, more effective preparation, and potentially, a higher success rate in international competitions. Moreover, knowing that elite Breakers tend to have a specific body type and composition could also refine talent scouting and selection processes, allowing for more targeted recruitment based on the physical profiles that correlate with higher performance levels in Breaking. However, it is deemed necessary to emphasize that the management of athletic talent is considered an extremely complex process, which should not be limited, in a predictive context, to merely evaluating a few basic anthropometric traits [6]. Additionally, considering the very intense repeated efforts (i.e., all-out form) that happen over the course of a Battle, more investigations related to Breaking’s physiological demands should also be conducted [60,61].

### 4.2. Study Limitations

Given the modest sample size and the differences in this pilot study, which focused on analyzing high-level or top-tier athletes, the external validity is inherently limited. This restriction affects the ability to generalize the findings broadly, particularly in the study concerning B-girls. However, the primary aim of our study was to ensure the highest level of internal validity, particularly in the data acquisition and processing procedures, to facilitate appropriate comparisons with other researchers engaged in similar lines of inquiry, including those in other countries (e.g., a meta-analysis on these topics, which could provide a greater understanding of the phenomena under investigation).

## 5. Conclusions

This research provided insights into the anthropometric measures, body composition data, and strength performances of Italian B-boys and B-girls, divided according to their level of performance into elite (international) and sub-elite (national) athletes. The findings revealed that Italian Breakers of both genders are characterized by low height and weight values and low percentages of body fat mass and display a balanced mesomorph somatotype profile (marked development of muscular mass). It was hypothesized that this may be due to Battles’ demands that emphasize selection procedures and training regimens favoring these specific body proportions, dimensions, and composition characteristics. Moreover, due to the low sample sizes, the results from the correlation analysis did not reveal any significant differences in the variables investigated between sub-elite- and elite-level Breakers. However, the findings obtained in this study act as a primary investigation and as a blueprint for further study on Breaking and are able to furnish professionals in the field with valuable information, aiding in their customization of specific training procedures and guiding initiation and selection processes within this sport.

## Figures and Tables

**Table 1:** Sample composition presented by gender and level of qualification.

Gender	Elite	Sub-Elite	
Male	20.8%	79.2%	100.0%
Female	33.3%	66.7%	100.0%
Total	24.2%	75.8%	100.0%

**Table 2:** General measures (mean (standard deviation)/[95% I.C.])/(Shapiro–Wilk W; *p*).

Variables	Female	Male	Welch’s *t*-Test
Height (m)	1.585 (0.076)	1.708 (0.057)	*p* = 0.001 *** d = 1.814
	[1.526–1.644]	[1.684–1.732]	
	(0.985; 0.985)	(0.970; 0.673)	
Weight (kg)	54.17 (6.31)	63.80 (6.58)	*p* = 0.002 *** d = 1.495
	[49.31–59.14]	[61.02–66.58]	
	(0.941; 0.593)	(0.954; 0.335)	
BMI (kg·m^−2^)	21.55 (1.72)	21.87 (2.17)	*p* = 0.663 d = 0.164
	[20.22–22.88]	[20.95–22.79]	
	(0.910; 0.317)	(0.946; 0.224)	

BMI = body mass index. *p* = *p*-value; *** highly significant; d = Cohen’s d (effect size); d > 1 = huge effect.

**Table 3:** Skinfold measurements and body girth and breadth values (mean (standard deviation)/[95% I.C.]/(Shapiro–Wilk W; *p*) and Welch’s *t*-test (female vs. male)).

Variables	Female	Male	Welch’s *t*-Test
Subscapular (mm)	10.24 (2.72)	8.21 (1.22)	*p* = 0.058 d = −0.967
	[8.15–12.33]	[7.70–8.72]	
	(0.712; 0.002)	(0.935; 0.125)	
Triceps (mm)	14.56 (2.65)	6.29 (1.64)	*p* < 0.001 *** d = −3.752
	[12.52–16.60]	[5.60–6.98]	
	(0.981; 0.970)	(0.934; 0.123)	
Midaxillary (mm)	9.33 (3,28)	4.48 (0,82)	*p* = 0.003 *** d = −1.885
	[6.81–11.85]	[4.48–5.17]	
	(0.908; 0.303)	(0.908; 0.032)	
Chest (mm)	5.84 (1.29)	3.91 (0.69)	*p* = 0.002 *** d = −1.867
	[4.84–6.84]	[3.62–4.20]	
	(0.817; 0.031)	(0.924; 0.073)	
Suprailiac (mm)	7.85 (2.48)	4.72 (0.98)	*p* = 0.005 *** d = −1.663
	[5.94–9.76]	[4.34–5.13]	
	(0.932; 0.502)	(0.869; 0.005)	
Abdominal (mm)	12.90 (3.02)	7.32 (1.86)	*p* < 0.001 *** d = −2.223
	[10.57–15.22]	[6.53–8.10]	
	(0.919; 0.383)	(0.947; 0.236)	
Thigh (mm)	23.27 (4.26)	9.14 (3.24)	*p* < 0.001 *** d = −3.733
	[19.99–26.55]	[7.77–10.51]	
	(0.976; 0.943)	(0.894; 0.016)	
Calf (mm)	16.20 (4.12)	7.47 (2.40)	*p* < 0.001 *** d = −2.586
	[13.02–19.37]	[6.46–8.48]	
	(0.920; 0.390)	(0.960; 0.438)	
Sum of 8 skinfolds (mm)	100.24 (17.06)	51.92 (10.63)	*p* < 0.001 *** d = −3.400
	[87,13–113.36]	[47.43–56.41]	
	(0.988; 0.993)	(0.963; 0.510)	
Bicep girths (cm)	26.50 (1.50)	29.66 (2.06)	*p* < 0.001 *** d = 1.759
	[25.35–27.65]	[28.80–30.53]	
	(0.948; 0.674)	(0.970; 0.662)	
Thigh girths (cm)	51.61 (2.82)	49.33 (4.55)	*p* = 0.098 d = −0.601
	[49.44–53.78]	[47.41–51.25]	
	(0.930; 0.479)	(0.916; 0.048)	
Calf girths (cm)	35.16 (1.66)	35.81 (3.82)	*p* = 0.505 d = 0.219
	[33.89–36.44]	[34.20–37.43]	
	(0.951; 0.696)	(0.725; 0.001)	
Humerus breadths (cm)	5.85 (0.33)	6.86 (0.36)	*p* < 0.001 *** d = 2.886
	[5.60–6.11]	[6.70–7.02]	
	(0.968; 0.880)	(0.943; 0.190)	
Femur breadths (cm)	9.13 (0.41)	9.71 (0.41)	*p* = 0.003 *** d = 1.412
	[8.82–9.45]	[9.53–9.88]	
	(0.894; 0.217)	(0.964; 0.534)	

*p* = *p*-value; *** = highly significant; d = Cohen’s d (effect size); d > 1 = huge effect.

**Table 4:** Body composition values (mean (standard deviation)/[95% I.C.]/(Shapiro–Wilk W; *p*) and Welch’s *t*-test (female vs. male)).

Variables	Female	Male	Welch’s *t*-Test
Body Fat-Free Mass (kg)	44.66 (5.28)	57.15 (5.15)	*p* < 0.001 *** d = 2.39
	[40.61–48.72]	[54.97–59.32]	
	(0.983; 0.977)	(0.967; 0.600)	
Body Fat (%)	17.54 (2.28)	10.28 (1.85)	*p* < 0.001 *** d = −3.49
	[15.79–19.30]	[9.50–11.06]	
	(0.969; 0.883)	(0.957; 0.387)	
Body Fat Mass (kg)	9.50 (1.74)	6.65 (1.73)	*p* < 0.001 *** d = −1.64
	[8.16–10.84]	[5.92–7.38]	
	(0.967; 0.866)	(0.950; 0.276)	

*p* = *p*-value; *** = highly significant; d = Cohen’s d (effect size); d > 1 = huge effect.

**Table 5:** Somatotype (mean (standard deviation)/[95% I.C.]/(Shapiro–Wilk W; *p*) and Welch’s *t*-test (female vs. male)).

Variables	Female	Male	Welch’s *t*-Test
Endomorphic	2.34 (1.07)	2.28 (0.90)	*p* = 0.886 d = −0.06
	[1.52–3.17]	[1.90–2.66]	
	(0.811; 0.027)	(0.854; 0.003)	
Mesomorphic	5.16 (1.00)	4.64 (1.39)	*p* = 0.241 d = −0.44
	[4.39–5.94]	[4.05–5.22]	
	(0.826; 0.040)	(0.959; 0.411)	
Ectomorphic	2.38 (0.92)	2.69 (1.28)	*p* = 0.451 d = 0.28
	[1.67–3.08]	[2.15–3.23]	
	(0.916; 0.359)	(0.974; 0.770)	

*p* = *p*-value; d = Cohen’s d (effect size); d > 1 = huge effect.

**Table 6:** Strength performance (mean (standard deviation)/[95% I.C.]/coefficient of variation %/(Shapiro–Wilk W; *p*)).

Variables	Female	Male
CMJ5—peak height (m)	0.279 (0.048)	0.401 (0.048)
	[0.238–0.319]	[0.381–0.421]
	17.3%	11.9%
	(0.856; 0.111)	(0.950; 0.264)
CMJ5—mean height (m)	0.258 (0.051)	0.367 (0.048)
	[0.215–0.301]	[0.346–0.387]
	19.6%	13.2%
	(0.833; 0.064)	(0.941; 0.168)
CMJ5—best contact time (s)	0.349 (0.051)	0.406 (0.076)
	[0.306–0.391]	[0.373–0.438]
	14.6%	20.37%
	(0.973; 0.922)	(0.975; 0.780)
CMJ5—mean contact time (s)	0.411 (0.047)	0.475 (0.059)
	[0.372–0.450]	[0.449–0.500]
	11.4%	12.4%
	(0.986; 0.987)	(0.973; 0.752)
CMJ5—peak power (w·kg^−1^)	26.07 (4.32)	32.18 (5.82)
	[22.45–29.84]	[29.72–34.64]
	16.6%	18.08%
	(0.975; 0.932)	(0.943; 0.193)
CMJ5—mean power (w·kg^−1^)	23.80 (3.88)	28.80 (4.21)
	[20.55–27.05]	[29.01–30.58]
	16.3%	14.61%
	(0.932; 0.533)	(0.943; 0.192)
Push-up—power (w·kg^−1^)	4.09 (1.09)	7.47 (1.55)
	[3.18–5.01]	[6.78–8.16]
	26.6%	20.7%
	(0.947; 0.681)	(0.980; 0.909)

CMJ5 = countermovement jump 5.

## Data Availability

The original contributions presented in the study are included in the article/Supplementary Materials, further inquiries can be directed to the corresponding author.

## Supplementary Materials

The following supporting information can be downloaded at https://www.mdpi.com/article/10.3390/sports12070197/s1, Table S1: Correlations among variables for males (B-boys); Table S2: Correlations among variables for females (B-girls).
