# Supplementary material for: Comparing the Anthropometrics, Body Composition, and Strength Performance of Male and Female Italian Breaking Athletes: A Pilot Study
Source: Sports (Basel). 2024 Jul 22;12(7):197. doi: 10.3390/sports12070197 (PMC11280681; doi:10.3390/sports12070197)
Supplement: Supplementary file 1 [file sports-12-00197-s001.zip › sports-3040691-supplementary.pdf]

Table S1 – Correlations among variables (b-boys)

		Age	Level	Weight	Height	BMI	Sum of 8 Skinfolds	Fat Body Mass (%)	Fat Body Mass(kg)	Body Fat Free Mass (kg)	Jump height	Contact time	Jump power	Push-up power
**Age**	Pearson Correlation	--												
	N	24												
**Level**	Pearson Correlation	0,049	--											
	Sig. (2-tailed)	0,822												
	N	24	24											
**Weight**	Pearson Correlation	**0,473***	-0,167	--										
	Sig. (2-tailed)	0,020	0,436											
	N	24	24	24										
**Height**	Pearson Correlation	0,074	0,124	0,342	--									
	Sig. (2-tailed)	0,732	0,565	0,102										
	N	24	24	24	24									
**BMI**	Pearson Correlation	**0,424***	-0,247	**0,779****	-0,320	--								
	Sig. (2-tailed)	0,039	0,245	0,000	0,128									
	N	24	24	24	24	24								
**Sum of 8 Skinfolds**	Pearson Correlation	0,143	0,073	0,325	-0,238	**0,484***	--							
	Sig. (2-tailed)	0,505	0,735	0,122	0,262	0,016								
	N	24	24	24	24	24	24							
**Fat Body Mass (%)**	Pearson Correlation	0,360	0,024	**0,674****	-0,055	**0,714****	**0,909****	--						
	Sig. (2-tailed)	0,084	0,913	0,000	0,799	0,000	0,000							
	N	24	24	24	24	24	24	24						
**Fat Body Mass(kg)**	Pearson Correlation	**0,458***	-0,044	**0,870****	0,116	**0,797****	**0,738****	**0,948****	--					
	Sig. (2-tailed)	0,025	0,837	0,000	0,590	0,000	0,000	0,000						
	N	24	24	24	24	24	24	24	24					
**Body Fat Free Mass (kg)**	Pearson Correlation	**0,451***	-0,198	**0,986****	0,398	**0,728****	0,167	**0,543****	**0,776****	--				
	Sig. (2-tailed)	0,027	0,353	0,000	0,054	0,000	0,435	0,006	0,000					
	N	24	24	24	24	24	24	24	24	24				
**Jump height**	Pearson Correlation	-0,154	-0,157	0,221	-0,030	0,209	-0,126	-0,071	0,026	0,276	--			
	Sig. (2-tailed)	0,495	0,484	0,324	0,893	0,351	0,575	0,753	0,910	0,214				
	N	22	22	22	22	22	22	22	22	22	22			

		Age	Level	Weight	Height	BMI	Sum of 8 Skinfolds	Fat Body Mass (%)	Fat Body Mass(kg)	Body Fat Free Mass (kg)	Jump height	Contact time	Jump power	Push-up power
**Contact time**	Pearson Correlation	0,233	-0,006	-0,177	0,092	-0,199	-0,005	-0,044	-0,106	-0,189	**-0,642****	--		
	Sig. (2-tailed)	0,296	0,979	0,432	0,683	0,375	0,984	0,845	0,637	0,399	0,001			
	N	22	22	22	22	22	22	22	22	22	22	22		
**Jump power**	Pearson Correlation	-0,173	-0,033	0,234	-0,043	0,224	-0,065	-0,004	0,088	0,270	**0,888****	**-0,903****	--	
	Sig. (2-tailed)	0,440	0,883	0,295	0,849	0,316	0,774	0,985	0,698	0,224	0,000	0,000		
	N	22	22	22	22	22	22	22	22	22	22	22	22	
**Push-up power**	Pearson Correlation	0,205	-0,078	0,161	0,028	0,152	-0,179	-0,104	-0,016	0,211	**0,716****	-0,168	**0,503***	--
	Sig. (2-tailed)	0,349	0,723	0,462	0,900	0,489	0,415	0,638	0,942	0,333	0,000	0,456	0,017	
	N	23	23	23	23	23	23	23	23	23	22	22	22	23

*** Correlation is significant at the 0.05 level (2-tailed) / ** Correlation is significant at the 0.01 level (2-tailed)**
Nominal variables considered: Level (Elite=1; Sub-elite=2)

Table S2 – Correlations among variables (b-girls)

		**Age**	**Level**	**Weight**	**Height**	**BMI**	**Sum of 8 Skinfolds**	**Fat Body Mass (%)**	**Fat Body Mass(kg)**	**Body Fat Free Mass (kg)**	**Jump height**	**Contact time**	**Jump power**	**Push-up power**
**Age**	Pearson Correlation	--												
	N	9												
**Level**	Pearson Correlation	-0,015	--											
	Sig. (2-tailed)	0,969												
	N	9	9											
**Weight**	Pearson Correlation	0,115	-0,658	--										
	Sig. (2-tailed)	0,768	0,054											
	N	9	9	9										
**Height**	Pearson Correlation	-0,053	-0,271	**0,765***	--									
	Sig. (2-tailed)	0,892	0,480	0,016										
	N	9	9	9	9									
**BMI**	Pearson Correlation	0,264	**-0,684***	0,631	-0,015	--								
	Sig. (2-tailed)	0,492	0,042	0,068	0,970									
	N	9	9	9	9	9								
**Sum of 8 Skinfolds**	Pearson Correlation	-0,274	0,318	0,248	0,129	0,239	--							
	Sig. (2-tailed)	0,476	0,404	0,521	0,741	0,536								
	N	9	9	9	9	9	9							
**Fat Body Mass (%)**	Pearson Correlation	-0,107	0,409	0,153	0,026	0,224	**0,968****	--						
	Sig. (2-tailed)	0,784	0,275	0,694	0,947	0,562	0,000							
	N	9	9	9	9	9	9	9						
**Fat Body Mass(kg)**	Pearson Correlation	-0,029	-0,086	**0,678***	0,435	0,541	**0,861****	**0,829****	--					
	Sig. (2-tailed)	0,942	0,826	0,045	0,242	0,133	0,003	0,006						
	N	9	9	9	9	9	9	9	9					
**Body Fat Free Mass (kg)**	Pearson Correlation	0,147	**-0,757***	**0,970****	**0,770***	0,575	0,011	-0,091	0,479	--				
	Sig. (2-tailed)	0,706	0,018	0,000	0,015	0,105	0,978	0,816	0,192					
	N	9	9	9	9	9	9	9	9	9				
**Jump height**	Pearson Correlation	-0,205	-0,475	0,209	0,167	0,089	-0,428	-0,550	-0,286	0,392	--			
	Sig. (2-tailed)	0,627	0,234	0,619	0,693	0,833	0,290	0,158	0,492	0,337				
	N	8	8	8	8	8	8	8	8	8	8			

		Age	Level	Weight	Height	BMI	Sum of 8 Skinfolds	Fat Body Mass (%)	Fat Body Mass(kg)	Body Fat Free Mass (kg)	Jump height	Contact time	Jump power	Push-up power
**Contact time**	Pearson Correlation	-0,305	-0,090	0,565	**0,722***	0,064	0,502	0,525	0,628	0,465	-0,142	--		
	Sig. (2-tailed)	0,463	0,832	0,144	0,043	0,880	0,205	0,181	0,095	0,246	0,737			
	N	8	8	8	8	8	8	8	8	8	8	8		
**Jump power**	Pearson Correlation	-0,022	-0,362	-0,091	-0,199	0,044	-0,602	**-0,711***	-0,537	0,111	**0,893****	-0,571	--	
	Sig. (2-tailed)	0,958	0,379	0,831	0,637	0,917	0,114	0,048	0,170	0,794	0,003	0,139		
	N	8	8	8	8	8	8	8	8	8	8	8	8	
**Push-up power**	Pearson Correlation	-0,310	-0,482	-0,293	-0,589	0,159	-0,506	-0,611	-0,549	-0,146	0,639	-0,431	**0,736***	--
	Sig. (2-tailed)	0,455	0,226	0,481	0,125	0,707	0,201	0,108	0,159	0,730	0,088	0,286	0,037	
	N	8	8	8	8	8	8	8	8	8	8	8	8	8

*** Correlation is significant at the 0.05 level (2-tailed) / ** Correlation is significant at the 0.01 level (2-tailed)**
Nominal variables considered: Level (Elite=1; Sub-elite=2)
